# Stroboscopic phenomena in superconductors with dynamic pinning landscape

**DOI:** 10.1038/srep14604

**Published:** 2015-10-01

**Authors:** Ž. L. Jelić, M. V. Milošević, J. Van de Vondel, A. V. Silhanek

**Affiliations:** 1 Departement Fysica, Universiteit Antwerpen, Groenenborgerlaan 171, B-2020 Antwerpen, Belgium; 2 Département de Physique, Université de Liège, Allée du 6-Août 17, B-4000 Liège, Belgium; 3 Institute for Nanoscale Physics and Chemistry, Department of Physics and Astronomy, KU Leuven, Celestijnenlaan 200D, B-3001 Leuven, Belgium

## Abstract

Introducing artificial pinning centers is a well established strategy to trap quantum vortices and increase the maximal magnetic field and applied electric current that a superconductor can sustain without dissipation. In case of spatially periodic pinning, a clear enhancement of the superconducting critical current arises when commensurability between the vortex configurations and the pinning landscape occurs. With recent achievements in (ultrafast) optics and nanoengineered plasmonics it has become possible to exploit the interaction of light with superconductivity, and create not only spatially periodic imprints on the superconducting condensate, but also temporally periodic ones. Here we show that in the latter case, temporal matching phenomena develop, caused by stroboscopic commensurability between the characteristic frequency of the vortex motion under applied current and the frequency of the dynamic pinning. The matching resonances persist in a broad parameter space, including magnetic field, driving current, or material purity, giving rise to unusual features such as externally variable resistance/impedance and Shapiro steps in current-voltage characteristics. All features are tunable by the frequency of the dynamic pinning landscape. These findings open further exploration avenues for using flashing, spatially engineered, and/or mobile excitations on superconductors, permitting us to achieve advanced functionalities.

A type-II superconductor can allow penetration of external magnetic field in terms of vortices, quantized filaments of magnetic flux inside which superconductivity is locally depleted. The whirl of superconducting current around this topological defect, responsible for its name, interacts with an applied electric current causing the motion of vortices in a manner analogous to the Lorentz force^1^. Unfortunately, this dissipative motion results in progressive Joule heating and quenching of superconductivity.

Over the past decades, the main strategy to prevent (or at least decrease) the described energy dissipation was to artificially anchor the vortices, using specifically designed arrays of pinning centers. In that respect, many alternatives were employed—e.g. pinning centers produced by irradiation with heavy ions^2^, chemically grown defects^3^, nanostructured perforations^4^, or permanent nanomagnets^5^. Regardless of the exact nature of pinning, the common idea has always been to create a spatial inhomogeneity in the superconducting condensate, i.e. locally suppress superconductivity on a scale comparable to the size of the vortex core. If then a periodic arrangement of the pinning centers is made, the superconductor can sustain a particularly high critical current at so-called matching fields, where the ideally homogeneous lattice of vortices is interlocked with the periodic lattice of the pinning centers^6^,^7^. Properties of such periodical pinning landscapes have been extensively investigated in the literature, with particular attention on the appearance of resonant features in the presence of the ac force^8^,^9^,^10^,^11^,^12^.

To date, all used pinning strategies involved an energy landscape imposed on permanent basis—i.e. neither its intensity nor its spatial distribution could be further modified. The question arises—how would a dynamic pinning landscape affect the superconducting properties? Clearly, the added degrees of freedom in terms of frequency and strength of the pinning, as well as its possible mobility and speed, next to its spatial geometry, could trigger new phenomena unattainable with static pinning. Such a study is not only interesting but also timely, since such dynamic and highly controllable pinning potential can now be achieved, for instance, by inhomogeneous light distribution (created by arrays of lasers^13^ or plasmonic nanostructures^14^,^15^,^16^) or by intensity modulation of the laser light.

The interaction of light with superconductivity has been of interest for decades, and it is now very well established that in most cases it leads to local heating and depletion of the superconducting condensate (a feature employed in superconducting single-photon detectors^17^,^18^,^19^,^20^,^21^), though at frequencies close to the superconducting gap light can interfere with the recombination of Cooper-pairs and even enhance superconductivity^22^. Interaction of laser light with vortices has also been studied, to form the base of the modern imaging technique of low-temperature laser scanning microscopy (LTLSM)^23^. However, light as a source of a time-dependent pinning potential in superconductors has not been explored to date, although it has been utilized for dynamic optical scanning in Bose-Einstein condensates (BECs)^24^, and for flashing ratchets in colloidal systems^25^,^26^,^27^,^28^,^29^,^30^,^31^.

Therefore, in this work, we investigate the fundamental consequences of a temporally periodic pinning landscape imprinted on a superconducting condensate. As a simple but exemplary case, we consider a superconducting stripe with longitudinally applied current, in magnetic field perpendicular to its plane, and with an oscillating depletion line along its middle (depicted in Fig. 1). We focus on the range of currents and fields where vortex motion induces a ‘resistive’ state^32^,^33^,^34^, and show the rich emergent phenomena as a function of the frequency of the pinning landscape.

## Results

What phenomenology should one expect from the frequency dependence of the pinning landscape? Clearly, if the change between the ON and OFF state of the pinning potential (further denoted as period *τ*) is faster than the characteristic relaxation time of the superconducting order parameter (i.e. *τ* ≤ *τ*_*GL*_, where *τ*_*GL*_ is the Ginzburg-Landau relaxation time, see Methods), then a recovery of the superconducting condensate is not possible—leading to permanent depletion and effectively static pinning. When the frequency of pinning oscillations is below this ultrafast limit, but still faster than the vortex velocity (or in terms of the characteristic time scales, 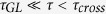, where *τ*_*cross*_ is the average time needed for a vortex to cross the pinning-free sample at certain magnetic field and applied current), there will be a noticeable change. Even though superconductivity has sufficient time to fully recover over one pinning cycle, the duration of the ON state is still not long enough to fully trap the moving vortex, hence the interaction between the flux quanta and the dynamic pinning can be considered weak (we refer to this regime as ‘vortex tapping’). At further decreased pinning frequencies, where *τ* becomes comparable with *τ*_*cross*_, one reaches the ‘vortex pinning’ regime where the motion of the flux quanta is greatly influenced by the variations in the pinning landscape. This is the richest part of the frequency phase diagram, and the main focus of our study. Finally, in the limit where *τ*→∞, the system becomes a simple alternation of two long-lasting states: the one where the pinning is OFF and the structure behaves as the pinning-free sample, and the second one with the pinning ON behaving as the static case extensively studied in literature.

### Stroboscopic resonances

**Figure 1:**
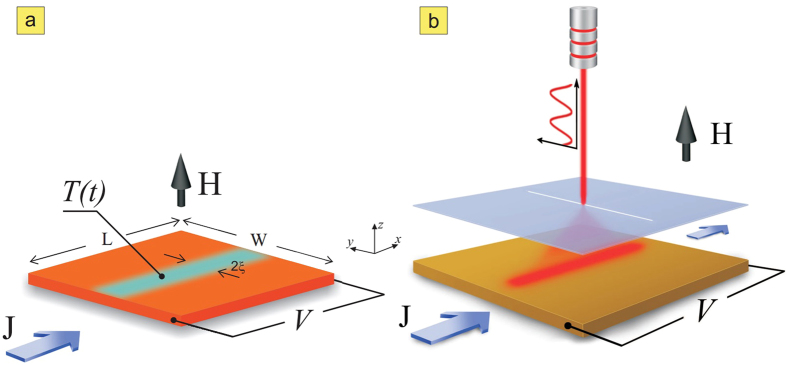
Layout of the studied system. (**a**) A superconducting stripe of width *W* with a central, time-dependent, pinning line of width comparable to the vortex size (*ξ* is the superconducting coherence length), and a 4-point probe applied *dc* current (with density *J*), and measured voltage (*V*, at contacts separated by distance *L*). A depletion region is simulated as sinusoidally oscillating local heating up to the critical temperature *T*_*c*_ and back to working temperature, with frequency *ω* (see Methods). (**b**) Possible experimental setup corresponding to (**a**), by using a laser light at the far field passing through a metallic mask to create time-dependent and spatially modulated depletion of the superconducting condensate.

**Figure 2:**
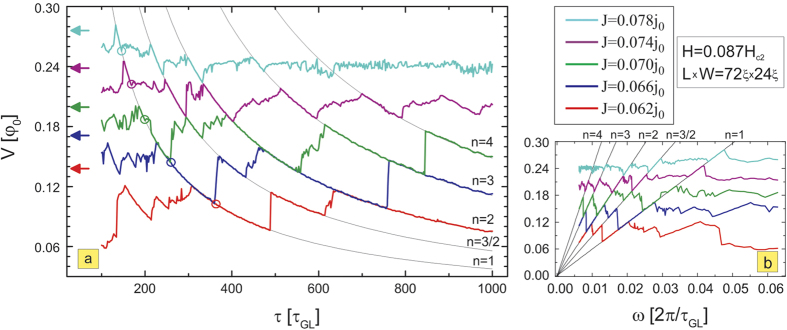
Voltage resonances. (**a**) Voltage is plotted versus the period *τ* of the oscillating pinning landscape shown in Fig. 1, for a given sample size and magnetic field, and for five different values of the applied current. Arrows indicate the voltage in absence of pinning for the corresponding current, while dots indicate the transition between two dynamic regimes: vortex tapping at low *τ* and vortex pinning at high *τ*. The black lines highlight the resonances where 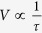 as further shown in (**b**) where voltage is plotted against pinning frequency *ω* = 2*π*/*τ*, and clearly shows a linear dependence when resonance conditions are met.

**Figure 3:**
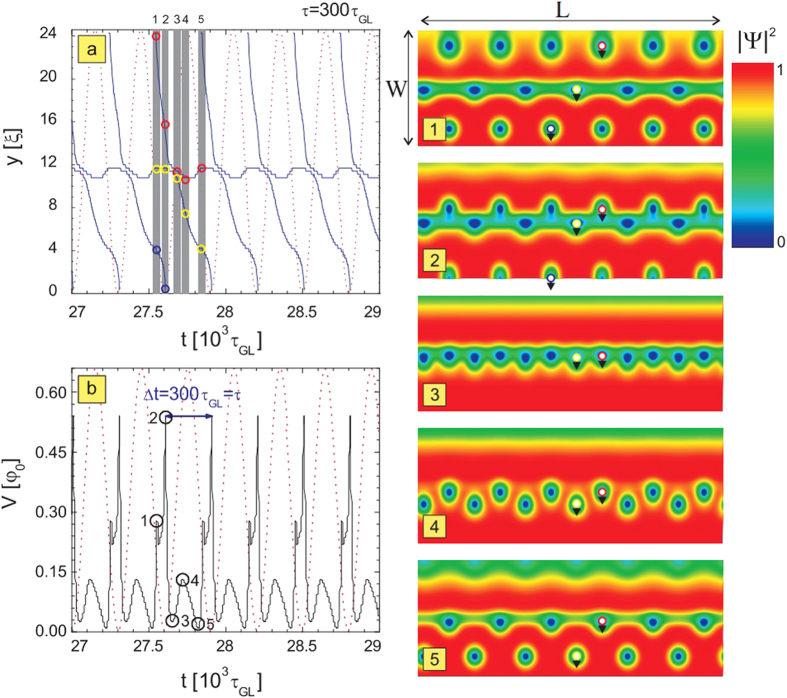
Vortex trajectory and temporal voltage response at the first resonance. The location of the vortex core traveling across the sample as a function of time (**a**), and the corresponding change in the voltage (**b**), for one example of the system at the first resonance (in particular, for *J* = 0.066*j*_0_ and 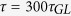, see Fig. 2). Dotted lines show the profile of the time-dependent pinning potential, where minima of the dotted lines represent the ON state of the pinning, and the maxima represent the OFF state. Points 1–5 denote one cycle of the vortex dynamics, marking the instances when the new vortex row enters (cycle begins) (1), some of the the previously existing vortices exit (2), the vortex row is trapped in the depletion region (3), the remaining preexisting vortices are depinned from the depletion region (4), and the remaining previously existing vortices exit the sample (5). Corresponding snapshots of the Cooper-pair density for states 1–5 are given in the right panel, showing rows containing six vortices in parallel and simultaneous motion. The resulting periodicity of the voltage during vortex motion exactly matches the period of the pinning, and entering/exiting vortices provide a flux change of exactly one flux-quantum per vortex row during that period.

In what follows, we study in detail the regimes where pinning landscape and moving vortices interact most. In Fig. 2, we present the diagram of the calculated voltage on the sample (with taken size *L* × *W* = 72*ξ* × 24*ξ*, sample is periodic in *L*, see Fig. 1 and Methods) as a function of the period of the pinning oscillations *τ*, varied between 100 and 1000*τ*_*GL*_. The voltage is a measure of dissipation for the given current, hence is intimately related to the vortex motion. Fig. 2(a) reveals clear indications of resonant behavior, namely, all *V*(*τ*) characteristics show well-defined occurrence of extendedly stable states perfectly and continuously linking the curves for different applied currents. This resonant behavior at different currents is highlighted by black lines in Fig. 2(a), and follows 1/*τ* dependence as evidenced in Fig. 2(b). To see the origin of this behavior, we chose one point on the curve (for *τ* = 300*τ*_*GL*_ and applied current density *J* = 0.066*j*_0_, where *j*_0_ is the unit of the current, see Methods) and monitored the vortex motion *y*(*t*) and voltage as a function of time (shown in Fig. 3(a,b)). For the chosen length of the simulation region *L* and the considered magnetic field, we actually had *N*_*v*_ = 6 parallel vortices simultaneously moving in a single row, as shown in the contourplots of the Cooper-pair density in the right panel of Fig. 3. There, points 1–5 are used to denote one period of the vortex dynamics, marking the characteristic instances: the entry of a new vortex row (beginning of the cycle—1), the exit of a single row of the previously present vortices (2), the trapping of the vortex row in the depletion region, together with remaining preexisting vortices (3), depinning of the second row of the previously present vortices from the depletion region (4), and finally, exit of the last preexisting vortices (5). As one can observe in Fig. 3, at the chosen resonance the frequency of pinning is exactly synchronized with the vortex motion—the period of the pinning matches exactly the period of the measured voltage (see Fig. 3(b)), and during one period effectively one vortex row crosses the sample from one edge to another, while one vortex row remains pinned all the time (see Fig. 3(a)), or in words of Faraday’s law—the voltage corresponds to the change of magnetic flux over period *τ* of exactly one flux quantum, multiplied by *N*_*v*_. For more information, we refer the reader to full animated data and description of vortex dynamics at resonances in the supplementary materials.

Therefore, we revealed a temporal matching effect which is in essence stroboscopic, i.e. caused by synchronization between pinning and vortex dynamics. Multiple resonances are possible, depending on the number of vortices that participate in the characteristic dynamics during one period of the pinning oscillations (further denoted by *n*). Every integer number *n* leaves the resonant fingerprint on the *V*(*τ*) characteristics, but also fractional resonances are possible—for example for *n* = 3/2, where 3 magnetic flux quanta (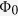) cross the sample over a period of 2*τ* (per vortex row). At all resonances, the voltage exhibits *V* ∝ 1/*τ* behavior (specifically 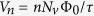, i.e. 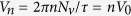 in dimensionless units, see Methods), hence it has linear dependence on pinning frequency, as shown directly in Fig. 2(b). Please note that in realistic experimental conditions, and significantly longer samples than shown in our simulation, the number *N*_*v*_ of simultaneously moving vortex rows at the resonance will be larger, hence the measured voltage at resonance will be proportionally larger than shown in our results—which facilitates the observation of the reported phenomenon.

The initiated reader will immediately notice the resemblance of this temporal matching events to the spatial commensurability phenomena employed in the past to enhance the critical parameters of superconductors. In our case, the system is always in a dynamic regime, but matching effects in resonances do decrease the voltage, hence also decrease the overall dissipation. Looking at the *n* = 1 resonance in Fig. 2, we note the possible tuning of resistance in a very broad range by simultaneous adjustment of *τ* and applied current—both externally controllable parameters.

### Shapiro steps

**Figure 4:**
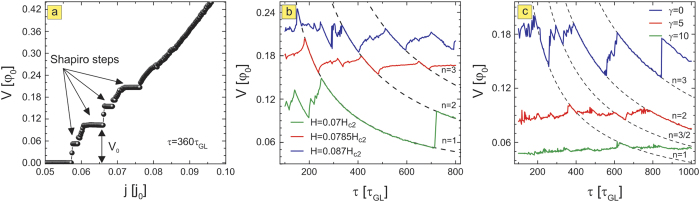
Shapiro steps. (**a**) The Shapiro steps in *V*(*j*) characteristics for the pinning period of 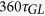. *V*_0_ indicates the Shapiro step at first resonance. (**b**) The resonant behavior in *V*(*τ*) for different values of magnetic field indicates possible Shapiro steps in magnetoresistivity. (**c**) The resonant behavior in *V*(*τ*) is also found for varied disorder, i.e. varied inelastic phonon-electron scattering time, with effective influence on the viscosity of the superconducting condensate (parameter *γ* in the theory, see Methods).

Another careful look at the resonances shown in Fig. 2 reveals more important details. Particularly, we observe that voltage curves obtained for different currents can overlap at certain pinning periods. In other words, for specific *τ* the system can exhibit identical voltage at two different bias currents. One of such cases is examined in Fig. 4, where we constructed the current-voltage *J*–*V* characteristics for 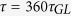. For *J* = 0.062 − 0.066*j*_0_ the system remains in the same *n* = 1 resonance, as seen in Fig. 2, and consequently *J*–*V* characteristics show a Shapiro step at these currents. At larger currents more Shapiro steps are found, corresponding to higher resonances and exhibiting *exactly quantized voltages*—equal to *nV*_0_, thus tunable by *τ*.

It is actually a well-known fact that when a superconductor in the presence of periodic potential is driven with the superimposed *dc* and *ac* force, Shapiro steps can be experimentally found in the *I*–*V* characteristics^35^,^36^,^37^,^38^. In our case Shapiro steps appear due to fact that we apply *dc* Lorentz force via magnetic field and current, and on the other side the time-dependent pinning potential is contributing with the *ac* component. The presence of the pinning potential reflects in the variation of the superconducting order parameter, which then translates in the *ac* variation of the supercurrent. In addition, due to the stripe-like profile of the depletion region, our structure is a time-dependent Josephson junction (*S* − *N*(*t*) − *S* junction), hence the relevance of Shapiro physics is clear.

Interestingly, in the reported investigation we observe Shapiro steps not only in current-voltage characteristics, but also with varying practically any parameter instead of current. An analogy can be drawn here with the case of static periodic pinning and an ac excitation (both for superconducting vortices and colloids), for which resonant features have been reported^8^,^10^,^12^,^25^. In our case, the reason is that the origin of the stroboscopic phenomenon is the synchronization of vortex motion and time-dependent pinning, and vortex motion can be influenced in more ways than just by current. In Fig. 4(b) we demonstrate the dependence of the *V*(*τ*) characteristics on the applied magnetic field, for fixed applied current. We again note the presence of the resonances, which obey the exact same 1/*τ* behavior as in Fig. 2(a). This leads us to the conclusion that Shapiro steps should also be expected in *V*(*H*) characteristics, i.e. in magnetoresistance. For additional check, we conducted simulations for varied inelastic phonon-electron scattering time of the superconductor, such that the effective viscosity for vortex motion changes. Even then resonances are observed to follow the same 1/*τ* behavior and where, with increased viscosity (parameter *γ* in the theory, see Methods), the vortices slow down, and voltage slides down on the resonant curve (see Fig. 4(c)). Further relations between vortex velocity and voltage will be explored in the Discussions section.

## Discussion

### Voltage-phase relation

**Figure 5:**
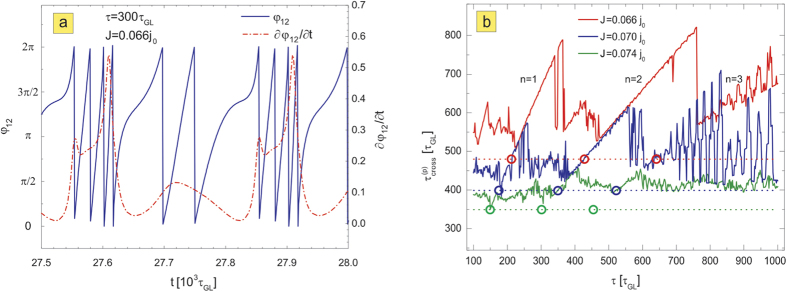
Relation of voltage to phase and vortex velocity. (**a**) The temporal evolution of the phase difference (blue line) and the temporal derivative of the phase difference (red dash-dots) measured between the voltage contacts, for the situation corresponding to Fig. 3. (**b**) The crossing time of one vortex as a function of the pinning period *τ*, for different applied current. Horizontal dashed lines represent the vortex crossing time in the pinning-free sample. Open dots indicate *τ* = τ^*^, the period of the pinning for which the vortex crossing time becomes the same as in the absence of pinning (consequently, the corresponding voltages match, c.f. Fig. 2).

In a region where there is no interconversion between supercurrent and normal current densities (i.e., wherever **∇·j**_**s**_ = 0, thus well outside moving vortex cores) the electric field **E** and the gauge-invariant vector potential **A**_S_ are related by the simple gauge-independent equation


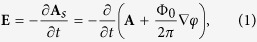


where **A** is the vector potential within a chosen gauge, and *φ* is the phase of the order parameter in the same gauge. Our calculations show that the contribution of ∇*φ* to **A**_**s**_ is far larger than that due to **A**. This argument arises from the fact that the magnetic-flux contributions can be neglected relative to kinetic-energy contributions when the linear dimensions of the nanocircuits under consideration are small by comparison with the Pearl length^39^. For this reason, at least for relatively narrow nanocircuits it is an excellent approximation to write


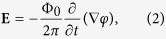


hence


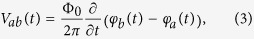


which is the standard Josephson relation between voltage and phase (*a* and *b* being the arbitrary points between which the voltage is measured). For additional check, we plotted in Fig. 5(a) the change of phase *φ*_12_ =*φ*_2_ − *φ*_1_ between the points where the voltage is measured, together with the time derivative of *φ*_12_, and confirmed that 
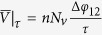
, as stipulated by Eq. (3). As also shown in e.g. Ref. 40, the *dc* average of *V*(*t*) during vortex crossing will be *V*_*dc*_ = Φ_0_*R*, where *R* is the rate of vortex crossings. The latter corresponds exactly to our resonant conditions.

### Effects on vortex dynamics and velocity

The description of the vortex dynamics corresponding to Fig. 3, and the higher resonances involving more vortices is presented in the Supplementary Materials. Essentially, the voltage associated with vortex crossing *V*_*ab*_(*t*) is proportional to the vortex velocity *v*(*t*) (
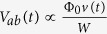
, where *W* is the width of the sample), and in the case of multiple crossing vortices the total voltage is the sum of their individual contributions. Knowing this, and looking at the time-averaged voltage shown in Fig. 2, one can establish relations between time-averaged vortex velocities at different currents, with or without pinning.

Usually, in experiments the vortex velocity can only be reliably estimated at high magnetic field^41^,^42^,^43^, since vortex-vortex interaction dominates over vortex-pinning and therefore the velocity distribution function is very narrow. In our numerical simulations we tracked the velocities of individual vortices, by developing a software capable of mapping the vortex trajectory in real time (shown in Fig. 3(a)). From it, we can also obtain the average crossing time in the presence of the pinning, 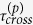, which includes the time vortices spend trapped in the flashing pinning potential. At the *n*th resonance, the average time a vortex spends in motion during 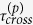 is exactly *τ*/*n*, and the average vortex velocity during its motion is therefore 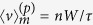. The time a vortex spends pinned is also approximately *τ*/*n*, as can be seen in Fig. 3(a) and the Supplementary Material. Hence, at resonance, 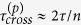. This is indeed verified in Fig. 5(b), where we show the dependence 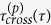, corresponding to the cases considered in Fig. 2. Therefore, if voltage is measured experimentally for varied *τ* and applied current, one can establish where voltage in absence of pinning (indicated by arrows in Fig. 2) meets the *V*(*τ*) curve during the resonance, and determine τ^*^ for which time-averaged vortex velocities with and without pinning are the same, i.e. 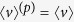, hence 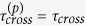. Then, the time-averaged vortex velocity without pinning can be conveniently estimated in experiment as 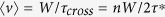. Remarkably, for pinning flashing with period *τ* = τ^*^, the average vortex velocity when in motion 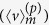 will be *twice larger* than the average vortex velocity in absence of pinning.

Here we provide some estimates of the relevant quantities for NbN frequently used for superconducting single-photon detectors^44^. Taking *T*_*c*_ = 12.7 K and the working temperature *T* = 5.5 K, and coherence length *ξ*(0) = 4 nm, we estimate 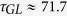 fs and upper critical field 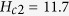 T. Since *τ*_*cross*_ is more than hundred times the *τ*_*GL*_, this translates into *τ*_*cross*_ ≥ 10 ps. Range of the considered pinning periods *τ* for this particular case goes from 7.2 ps up to 72 ps, which means that the highest considered pinning frequency is 140 GHz. If the width of the sample is increased, all of the resonances are shifted towards higher *τ*, making them feasible at frequencies below 20 GHz. These values are comparable with the parameters of mode-locked lasers where pulse duration goes up to 5 fs, and with achievable repetition rates of 100 GHz^45^. Expected value of the resonant voltage *V*_0_ is of the order of 10–100 *μ*V. A depleted region of several *ξ* could be imprinted using the far field excitation of a slit graved in a thick metallic mirror as schematically shown in Fig. 1(b). However, in case the size of the depleted region should be smaller than the wavelength of the impinging light, it would be necessary to work in the optical near-field.

Finally, we take a step back to the case of faster oscillations of the pinning, the so called ‘tapping’ mode. The reason is that a careful look at Fig. 2(a) reveals the possibility of having a *larger* voltage at the resonance (see beginning of the *n* = 1 curve) than in the case without any pinning in the sample (indicated by an arrow in the vertical axis). This is highly counterintuitive, as adding pinning to superconductors is supposed to anchor vortices and decrease dissipation. This unusual feature is only possible in the tapping regime (see the dots separating the tapping from the pinning regime in Fig. 2). Namely, the conditions to obtain this phenomenon are such that when the vortex enters the sample, the pinning is at its maximum, but weakens as vortex approaches. Therefore, the attractive force between the vortex and the depletion region will accelerate the vortex, only to tap it and release as if there is no pinning. Hence the average vortex velocity is larger than in the case without pinning, which is a unique case of pinning-enhanced dissipation.

In summary, we presented the very first consideration of the effects of dynamic pinning landscape on resistive state in superconductors. We revealed stroboscopic matching between vortex dynamics and pinning oscillations, which leaves unexpected signature as resonances in the measured voltage versus the period of the pinning, continuous in wider parameter space (for different applied current, magnetic field, microscopic sample parameters). Most of these parameters are externally variable, hence system can be easily tuned along the resonant condition, to achieve beneficial resistive conditions, Shapiro physics without any nanostructuring, or advanced control of flux quanta, all of which deserve further experimental and theoretical pursuit.

## Methods

In this work, the time-dependent Ginzburg-Landau (TDGL) equations are used to calculate the voltage as a function of the applied current, magnetic field, material parameters and the period of the time dependent pinning^46^,^47^. The TDGL model is known to provide excellent description of the superconductive dynamics close to the critical temperature *T*_*c*_. The TDGL equations we solved read:


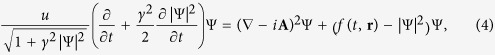



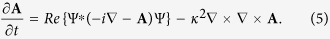


These equations are given in the gauge of zero electric potential, and are expressed in the dimensionless units, where the order parameter, Ψ, is given in the units of 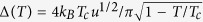 at temperature *T* (*k*_*B*_ is the Boltzmann constant, *u* = 5.79 is the ratio of the relaxation time of the order parameter phase and the relaxation time of the order parameter amplitude^46^,^48^), and the vector potential, **A**, is given in units of Φ_0_/2*πξ*(*T*) (Φ_0_ is the flux quantum). The parameter *γ* = 2*τ*_*in*_Δ(*T*)/*ħ* (*ħ* is the Planck constant) is determined by the product of the inelastic collision time *τ*_*in*_ for electron-phonon scattering and Δ(*T*), and *κ* is the Ginzburg-Landau parameter which is by its definition dimensionless. Time is expressed in the units of Ginzburg-Landau relaxation time, *τ*_*GL*_ = *πħ*/8*k*_*B*_(*T*_*c*_ − *T*)*u*, and all of the distances are scaled with the coherence length, *ξ*(*T*). The transport current was introduced via the boundary condition for the vector potential in the *y* direction: 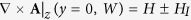, where 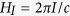 is the magnetic field induced by the current *I*. From the vector potential it is possible to obtain the voltage by using the 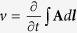. The applied current is given in units of 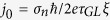 (*σ_n_* is the normal-state conductivity), the magnetic field in units of 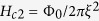, and the voltage scale is given by 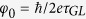. In these units, *j*_0_ can be related to the theoretical depairing current *j*_*dp*_, as 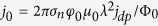, where *μ*_0_ is magnetic permeability of vacuum, and *λ* is the superconducting penetration depth.

In order to simulate a long sample, we apply periodic boundary conditions in the direction of the applied current, 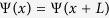 and **A**(*x*) = **A**(*x* + *L*), where *L* = 72*ξ* is the length of the simulated rectangular unit cell (with width *W* = 24*ξ*). In *y* direction the superconductor-vacuum boundary conditions were applied: 
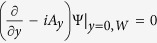
.

The function *f*(**r**, *t*) is the time-dependent pinning potential:


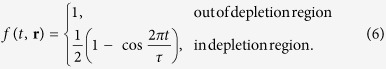


The term 2*π*/*τ* in Eq. (6) is the angular frequency *ω* of the pinning. This time-dependent pinning potential arises from temperature variation due to the local heating from some external source (e.g. laser setup). From there the temperature can be varied around its critical value, so we are able to create a region of depleted superconductivity in the middle of the sample. The function *f*(*t*, **r**) provides oscillations of the order parameter in the depletion region, which shifts between the superconducting state (*f* = 1), and normal state (*f* = 0). A relation between the temperature and *f*(*t*, **r**) is given through 
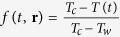
, where *T*_*w*_ is the superconductor working temperature outside of the depletion region. By adding the additional terms in the pinning potential *f*(*t*, **r**) it is also possible to account for the impact of the non-equilibrium quasi-particle distribution, where such distribution is calculated outside of TDGL theoretical frame^18^.

In order to properly describe the possible temperature oscillations in the sample, it is required to couple the heat-balance equation (HBE) to the existing TDGL model as it was done in the work of Vodolazov *et al.*^49^ This task is far from trivial since the characteristic thermal coefficients appearing in the HBE (thermal conductivity of the material, the specific heat and the heat removal through the substrate) are themselves temperature dependent. The case described in the present work corresponds to the situation where heat removal is very efficient due to the negligible heat diffusion within the sample. By decreasing the heat removal through the substrate, eventually the order parameter oscillations in the depletion region will transform into a hot-spot where the order parameter will become strictly zero. Under these circumstances oscillations will be lost and so will be the Shapiro steps. Consequently, a purely bolometric effect will rule the behavior of the system, which may also serve as a pinning center, but the dynamic aspect will be simply lost. Notice also that HBE does not appear natively from Ginzburg-Landau theory, nor from the derivation of TDGL equations from BCS theory, and therefore we cannot determine the exact microscopic nature of the thermal coefficients at question.

Numerical simulations were all performed in the same manner, starting from the Meissner state (zero-field, zero-current cooled), then the magnetic field was applied, and subsequently current was gradually raised to the desired value over a period of 5000*τ*_*GL*_. Total duration of the simulation for each set of the parameters was the same, 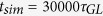.

In order to determine the vortex velocity, both local and average, we developed a vortex tracking software capable of locating the individual vortex and then tracing vortices in real time. As a result we simultaneously obtained information about the vortex paths, vortex crossing time and vortex velocity, for all participating vortices.

## Additional Information

**How to cite this article**: Jelić, Ž. L. *et al.* Stroboscopic phenomena in superconductors with dynamic pinning landscape. *Sci. Rep.* **5**, 14604; doi: 10.1038/srep14604 (2015).

## Supplementary Material

Supplementary Animation 1

Supplementary Animation 2

Supplementary Animation 3

Supplementary Information
