# Supplementary figures and images for: Stroboscopic phenomena in superconductors with dynamic pinning landscape
Source: Sci Rep. 2015 Oct 1;5:14604. doi: 10.1038/srep14604 (PMC4589687; doi:10.1038/srep14604)

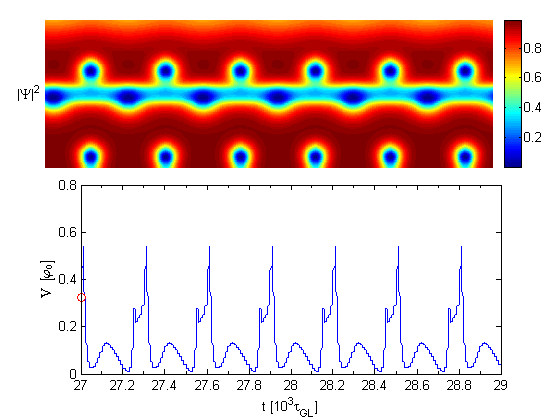

Supplement: Supplementary Animation 1 [file srep14604-s1.gif]

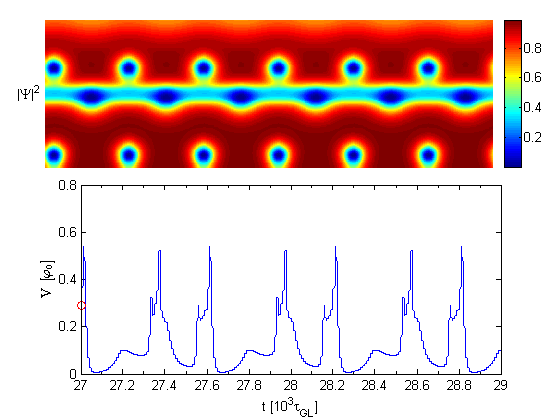

Supplement: Supplementary Animation 2 [file srep14604-s2.gif]

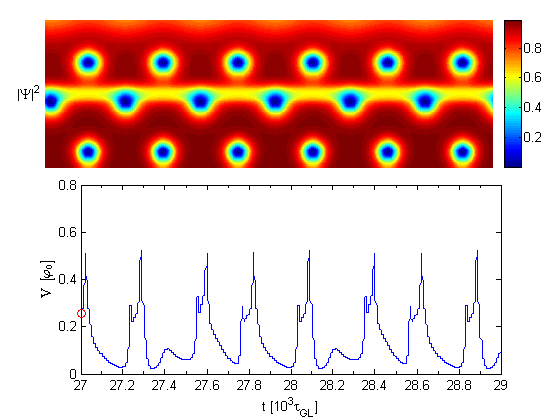

Supplement: Supplementary Animation 3 [file srep14604-s3.gif]
