# Supplementary material for: Stroboscopic phenomena in superconductors with dynamic pinning landscape
Source: Sci Rep. 2015 Oct 1;5:14604. doi: 10.1038/srep14604 (PMC4589687; doi:10.1038/srep14604)
Supplement: Supplementary Information [file srep14604-s4.pdf]

Supplementary material: Stroboscopic phenomena in superconductors with dynamic pinning landscape

Ž. L. Jelić,[1,2,*] M. V. Milošević,[1] J. Van de Vondel,[3] and A. V. Silhanek[2]

[1]*Departement Fysica, Universiteit Antwerpen, Groenenborgerlaan 171, B-2020 Antwerpen, Belgium*
[2]*Département de Physique, Université de Liège, Allée du 6-Août 17, B-4000 Liège, Belgium*
[3]*Institute for Nanoscale Physics and Chemistry, Department of Physics and Astronomy, KU Leuven, Celestijnenlaan 200D, B-3001 Leuven, Belgium*

[*] Correspondence to Ž. L. Jelić: zeljko.jelic@uantwerpen.be

I. VORTEX DYNAMICS AT STROBOSCOPIC RESONANCES

As stated in the main text, when dynamic pinning is introduced into the superconductor, recurring resonances may appear. These resonances are observable in the voltage (V) dependence on the pinning period (τ), as presented in Fig. 1 below (and Fig. 2 of the manuscript). In what follows we explain in more detail the interaction between the vortex motion and the dynamic pinning landscape at the pinning period values of τ =300, 600 and $800\tau_{GL}$, indicated by open dots on the first, second and third resonance in Fig. 1, respectively.

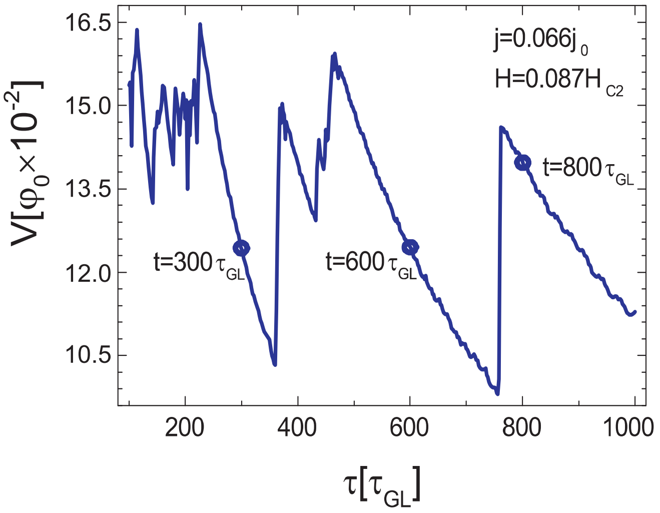

FIG. 1. **Voltage resonances.** Voltage as a function of the periodic time-dependent pinning potential, for given values of applied current and magnetic field. Open dots on the first, second and the third resonance indicate values of the pinning period for which a more in-depth analysis is presented.

In order to better understand the interaction between the superconducting condensate and the dynamic pinning during the first resonance, we selected one point in Fig. 1 at $\tau = 300\tau_{GL}$ and mapped the trajectories of the vortices $y(t)$ while moving across the sample. The trajectories are presented in Fig. 2(a) using blue lines, whereas the corresponding voltage versus time is shown in Fig. 2(b). In Fig. 2 we also show the dynamic pinning strength as a dotted line. Each of the minima in the pinning potential corresponds to the ON state of pinning, while the maxima relate to the OFF state of pinning. It is evident from this figure that during one pinning period only one vortex enters the sample, just after the transition between the OFF and ON states. One instance of vortex entry is denoted with 1 on Fig. 2(a). The entry is accompanied by a voltage peak c.f. Fig. 2(b). Almost immediately after this event, part of previously present vortices that were closest to the edge exit the sample (instance 2). This is accompanied with adjacent peak in voltage (Fig. 2(b)). The vortices subsequently travel towards the central part of the sample where the depletion region is located, and while doing so they interact with both the depletion region and the rest of the preexisting vortices still present in the sample. The pinning of the vortex causes the minimum of voltage (instance 3). Between the ON and OFF state transition, while the vortex row is temporarily trapped, the remaining previously existing vortices are depinned from the depletion region (instance 4). Eventually, all of the preexisting vortices are moving towards the edge (instance 5), and leave the sample. The explained vortex dynamics can be regarded as the state in which effectively one vortex row always moves, while another is always trapped in the depletion region. The file SupplementaryAnimation1.gif contains an animation summarizing this periodic motion as described above.

To exemplify the second resonance we select the point $\tau = 600\tau_{GL}$ from Fig. 1. The corresponding map of the vortex motion and the time dependent voltage are given in Fig. 2(c) and (d), respectively. In this case two vortex rows participate in the characteristic dynamics during one cycle. When the pinning state is OFF, the first vortex row enters (with trajectory shown as green line on Fig. 2(c)), accompanied by a peak in voltage. Under the influence of the pinning, the vortex row remains in the depletion region, while another row enters the sample (blue line on Fig. 2(c)). Temporary, both rows are trapped by the pinning, after which they leave the sample, in a first-in first-out manner. The related animation of vortex motion is provided in the file SupplementaryAnimation2.gif.

As discussed in main text of the manuscript, the number of vortex rows that participate in the characteristics dynamics during one pinning cycle progressively increases as we go to higher resonances. The final subset of figures in Fig. 2 ((e),(f)) presents the vortex dynamics for $\tau = 800\tau_{GL}$, i.e. at the third resonance of Fig. 1. Here, with colors blue, green and purple we denote trajectories of three vortex rows that appear during one cycle of the dynamic

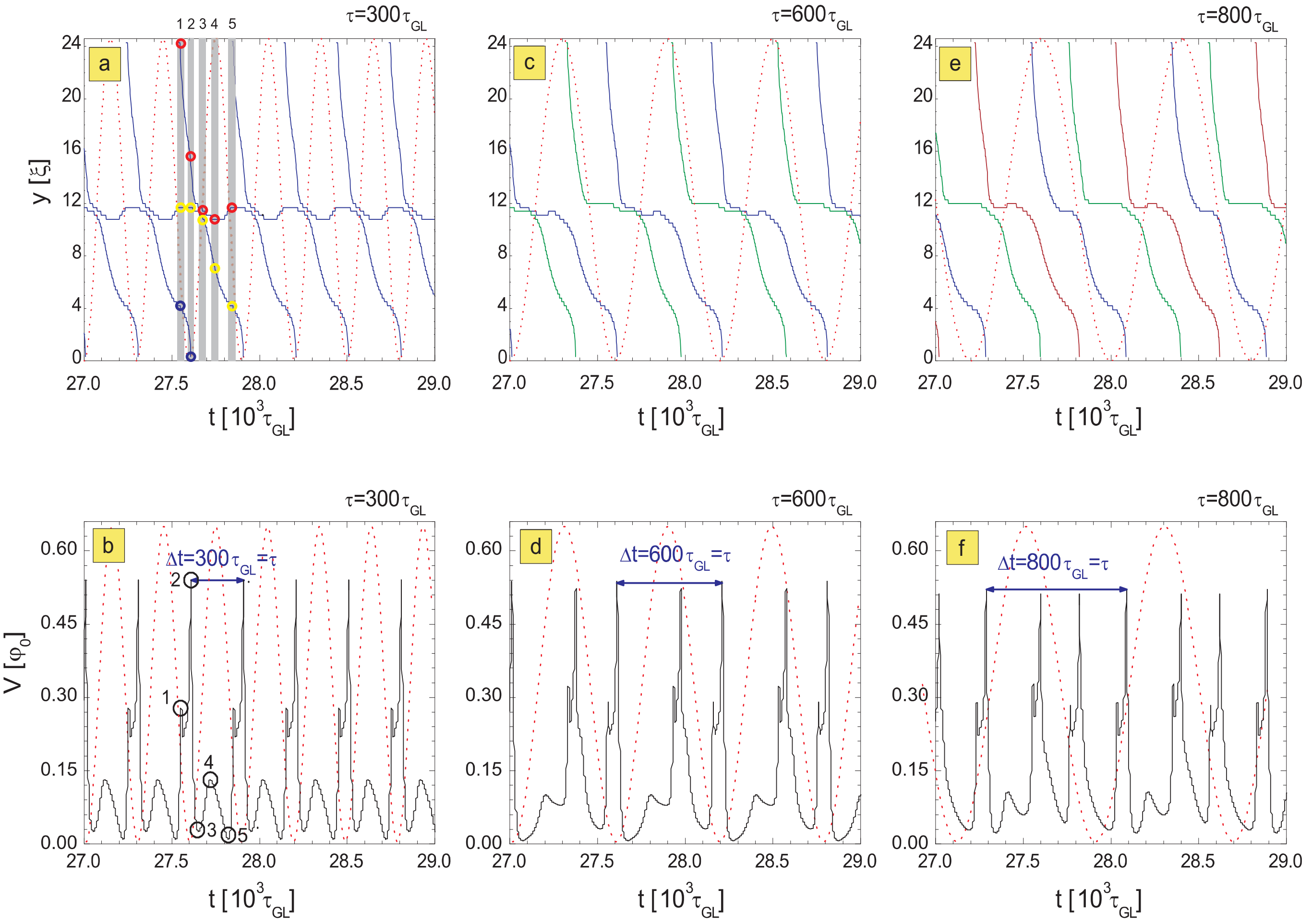

FIG. 2. **Vortex trajectories and voltage versus time during the resonance.** (a),(c),(e) The trajectory of the vortices transiting through the sample as a function of time, for the first, second and third resonance shown in Fig. 1 (τ =300, 600 and 800τ_{GL}, respectively), and the corresponding voltages (b),(d),(f). Dotted lines represent the time-dependent pinning potential, where minima correspond to the pinning state ON, and the maxima are the pinning state OFF. Different colors of the vortex trajectories indicate that two (green and blue on (c)) or three (green, blue, purple on (e)) vortices participate in characteristic dynamics during one pinning period. Points 1-5 in (a) and (b) mark important instances during one cycle of the periodic vortex dynamics, indicating the new vortex row entry (begining of the cycle), first row of the preexisting vortices leaving, the vortex row pinned, the remaining preexisting vortices leaving the depletion region, and the second row of preexisting vortices reaching the edge of the sample (end of the cycle), respectively.

period. The voltage correspondingly shows triplets of maxima and minima appearing over the cycle. The situation is illustrated in the animation SupplementaryAnimation3.gif.

In case where some vortices simultaneously appear as parallel pairs, triplets, etc. their $y(t)$ trajectories are identical, and different colors of trajectories would represent different vortex groups entering the sample at a given instances. The total voltage given in the $V(\tau)$ characteristic will then be multiplied by a factor equal to the number of the simultaneously moving vortices.
